# CRISPR loci reveal networks of gene exchange in archaea

**DOI:** 10.1186/1745-6150-6-65

**Published:** 2011-12-21

**Authors:** Avital Brodt, Mor N Lurie-Weinberger, Uri Gophna

**Affiliations:** 1 Department of Molecular Microbiology and Biotechnology, George S. Wise Faculty of Life Sciences, Tel Aviv University, Ramat Aviv, 69978, Israel

**Keywords:** CRISPR, Lateral Gene transfer, Horizontal gene transfer, viruses, archaea, competence

## Abstract

**Background:**

CRISPR (Clustered, Regularly, Interspaced, Short, Palindromic Repeats) loci provide prokaryotes with an adaptive immunity against viruses and other mobile genetic elements. CRISPR arrays can be transcribed and processed into small crRNA molecules, which are then used by the cell to target the foreign nucleic acid. Since spacers are accumulated by active CRISPR/Cas systems, the sequences of these spacers provide a record of the past "infection history" of the organism.

**Results:**

Here we analyzed all currently known spacers present in archaeal genomes and identified their source by DNA similarity. While nearly 50% of archaeal spacers matched mobile genetic elements, such as plasmids or viruses, several others matched chromosomal genes of other organisms, primarily other archaea. Thus, networks of gene exchange between archaeal species were revealed by the spacer analysis, including many cases of inter-genus and inter-species gene transfer events. Spacers that recognize viral sequences tend to be located further away from the leader sequence, implying that there exists a selective pressure for their retention.

**Conclusions:**

CRISPR spacers provide direct evidence for extensive gene exchange in archaea, especially within genera, and support the current dogma where the primary role of the CRISPR/Cas system is anti-viral and anti-plasmid defense.

**Open peer review:**

This article was reviewed by: Profs. W. Ford Doolittle, John van der Oost, Christa Schleper (nominated by board member Prof. J Peter Gogarten)

## Background

**Figure 1:**
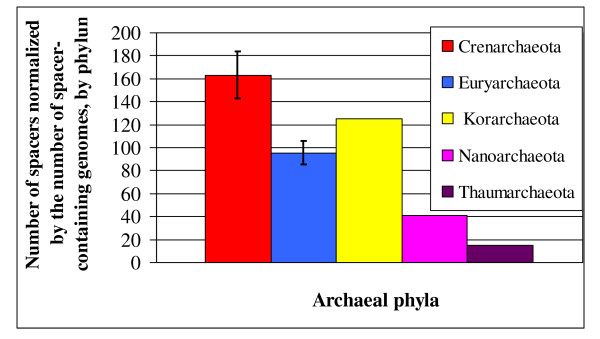
**Average number of CRISPR spacers per phylum, normalized by the number of spacer-containing genomes**. Error bars represent standard error of the mean.

CRISPR (Clustered, Regularly, Interspaced, Short, Palindromic Repeats)/Cas (CRISPR-associated) modules constitute acquired prokaryotic immune systems that protect prokaryotes against parasitic genetic elements, such as viruses [for recent reviews see [1-3]]. CRISPR/Cas systems contain repeated sequences that are interrupted by short non-repetitive DNA segments (20-50 bp long), termed spacers [4] (Figure 1). CRISPR arrays can be transcribed and processed into small crRNA molecules, which then lead to degradation of foreign nucleic acids by a mechanism based on complementary base-pairing [5,6]. CRISPR/Cas systems also have a mechanism that prevents targeting the locus encoding the CRISPR itself [7]. The systems are both adaptive, heritable and can be used to determine a history of past infections [7-9]

CRISPR elements can be found in almost all archaeal genomes and in approximately 40% of sequenced bacterial genomes [10]. Archaeal CRISPR loci tend to be larger than those found in bacterial genomes, most archaeal genomes having more than one CRISPR/Cas system [11]. Archaeal CRISPR/Cas has been shown to confer almost 100% immunity in cases where spacers were identical to the target sequence, but partial matches also provide substantial immunity in archaea [12]. A short seed sequence that requires a perfect match has been recently discovered in bacteria [6], but whether such seed also exists in archaea, remains to be determined. Archaea have unique viral parasites that probably cannot infect bacteria or eukaryotes. CRISPR/Cas systems can sometimes accidentally acquire an "auto-spacer", identical to a genome fragment of the CRISPR-containing organism itself, which can presumably lead to cell death [10]. One may speculate that the near-ubiquity of CRISPR/Cas in archaea implies that the benefit of having this form of defense outweighs the costs of the occasional autoimmunity event. Remarkably, no self-targeting spacers were found in archaea [[10], Sorek & Stern, personal communication]. Here we surveyed all archaeal spacers in order to gain insights into the mobile elements that infect archaea and how DNA is transferred among different species and strains.

## Results and discussion

### Crenarchaeota contain more CRISPR spacers

CRISPR/Cas systems are found across all archaeal phyla, yet CRISPR spacers are distributed unevenly among CRISPR-positive genomes (Figure 1). Despite the fact that much fewer crenarchaeal species have been sequenced, Crenarchaeal genomes have roughly the same number of spacers in the CRISPR database as Euryarchaeal species (Figure 1), having a higher average number of spacers per genome than genomes of the latter phylum (162.86 vs. 95.32, respectively, p = 0.003, Mann-Whitney-Wilcoxon test).

### Function and origin of spacer matches

**Figure 2:**
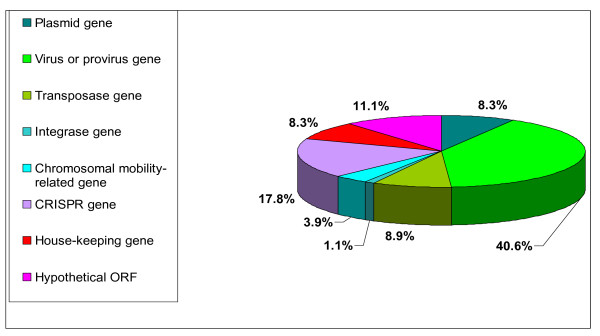
**The function distribution of the filtered hits according to the target gene, where the target is a coding sequence, or the gene closest to the sequence match**.

Predictably, nearly 50% of spacer hits were mapped to mobile genetic elements such as plasmids (Figure 2, Additional File 1 Table S1) and viruses (including proviruses) (Additional File 2 Table S2). Mobile element-associated genes, such as transposases (Additional File 3 Table S3), integrases (Additional File 4 Table S4), toxin-antitoxin systems and restriction-modification systems constituted (Additional File 5 Table S5) about 13% of spacer matches. Nevertheless, a substantial number of hits were found to match chromosomal genes, or non-coding regions in their close vicinity (Figure 2). These genes either had housekeeping functions (Additional File 6 Table S6), CRISPR-associated (Additional File 7 Table S7) or hypothetical ones (Additional File 8 Table S8). For example, five spacers in the *Methanococcus_vannielii *CRISPR array match ORFs encoding hypothetical proteins in chromosomes of *Methanococcus maripaludis *strains, and a spacer from *Halorubrum lacusprofundi *matched the sliding clamp PCNA of *Haloferax volcanii*. The vast majority of these ORFs (32/35) have no gene of putative viral function (e.g. tail or head protein, integrase, etc) in their neighborhood (5 genes upstream and 5 genes downstream). The presence of spacers matching house-keeping genes from related species indicates that the CRISPR/cas system often comes into contact with foreign DNA that is probably chromosomal in origin, primarily from other archaea, either by transduction, conjugation or transformation. For example, a chromosomal region can move to a natural plasmid by recombination and the resulting plasmid can subsequently be transferred by conjugation to a new cell, which will now recognize it as foreign. Under such circumstances, the CRISPR/Cas system could become activated and acquire a spacer directly-derived from plasmid, but originally being of chromosomal source. Alternatively, this could be attributed to viral activity, similar to generalized transduction in bacteriophages, where occasionally defective archaeal viruses package a portion of the host's DNA, resulting in a defective "transducing particle" which can enter an archaeal cell and similarly trigger the CRISPR/Cas system.

Curiously, frequent targets of CRISPR/Cas systems are *cas *(CRISPR-associated) genes belonging of other CRISPR/Cas systems (Figure 2) (Additional File 7 Table S7). This probably stems from the fact that CRISPR loci are often plasmid-encoded (Additional File 9 Table S9), but provides evidence that having one CRISPR/Cas system decreases the chances of acquiring additional such systems. Accordingly, several archaeal species that have multiple CRISPR loci [e.g. *Haloarcula marismortui *and *H. volcanii*, see [13]], have only a single set of *cas *genes.

### Archaea are not immune to CRISPR autoimmunity

Several cases of intra-genomic matches of spacers that could cause CRISPR-mediated autoimmunity [10] were identified in our survey. While the same spacer occurring twice, on the chromosome and on a plasmid, flanked by the same repeats, will be protected from degradation due to the 5' overlap of the repeat [7], a plasmid-located spacer providing an exact match to a sequence from a chromosomal gene would probably be cleaved. This is the case for *Halomicrobium mukohataei*, where the plasmid-located cas-less CRISPR array has repeats that are identical to the chromosomal one, implying that its spacers should be recognized by the chromosomal CRISPR/Cas system. One such plasmid encoded spacer, 39 bases long, is 100% identical to a sequence in a putative ORF in the chromosome, which should cause CRISPR "autoimmunity". The fact that this self-matching spacer is tolerated in this organism implies that either the proto-spacer associated motif (PAM) has been mutated in the chromosomal gene sequence rendering the gene immune [3] or that the chromosomal CRISPR/Cas system had become deactivated in this archaeon.

### Spacers reveal gene transfer events across species boundaries

**Figure 3:**
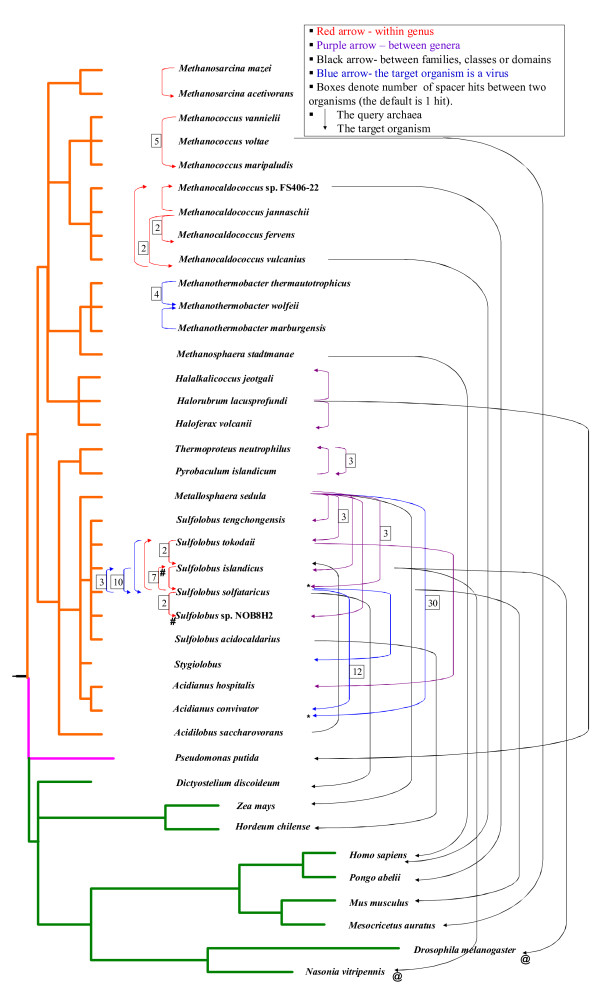
**A schematic phylogenetic tree of organisms that have contributed spacer sequences to archaea**. The direction of arrows points from the spacer towards its match. Red arrows, within genus matches; purple arrow, between genera matches; black arrows- matches between families, classes or domains; blue arrows, the match is to a virus known to infect that species (not including provirus sequences within genomes of cellular organisms). Boxes denote number of spacer hits between two organisms, absence of a box denoting a single match. "@", "#" equal best hits. Within species matches are not shown.

**Figure 4:**
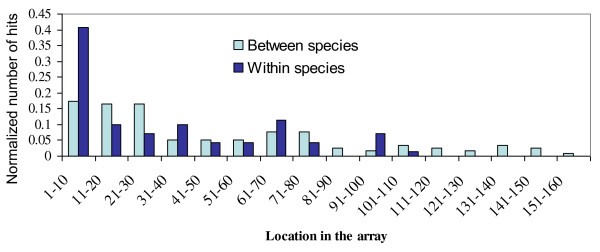
**Distribution of the fractions of between-species (light blue) and within species (dark blue) spacer hits according to their location in the CRISPR array**.

Normally, when examining gene transfer in microbial genomes one observes only genes that have been fixed, at least in a specific population of cells. In contrast, sequences found in CRISPR spacers can be used as indicators of whichever DNA the CRISPR-containing organism has encountered in some CRISPR/Cas-activation context, such as phage infection. Since CRISPR/Cas acquires DNA fragments that can be seen as "signatures" of foreign invaders, regardless of the function of the genes, spacers can tell us which sources of foreign DNA end up in the CRISPR-containing species. Most spacers have best matches outside the specific CRISPR containing species (Figures 3, 4, Additional File 10 Dataset 1), although usually falling within the same family of organisms. In some cases this probably reflects the existence of viruses that are not strictly species-specific, whereas other cases, where the hit is to a chromosomal gene, are more difficult to explain (see above). Obviously, in cases where the identity is not 100%, it may reflect exposure to a virus from the same viral family that has diverged in sequence, i.e. when viruses co-evolved/co-diverged with their hosts, or might reflect an instance where a virus is present in the database whereas the genome of the related virus from which the target-gene originated is not.

We observed 59 matches across archaeal genera (including 43 hits to archaeal viruses) and 10 matches to non-archaeal organisms (nine eukaryotes and one bacterium). Within the archaea, only a single cross-order/cross-family match was observed, in which a spacer from *Acidilobus saccharovorans *(order Acidilobales) was nearly identical to a sequence from an insertion-sequence (IS200/IS605 family OrfB protein) in *Sulfolobus islandicus *strain HVE10/4 (order Sulfolobales, see GI:323476134 and [14]). In contrast, many inter-genera matches were observed, especially cases where spacers from one genus matched a virus known to infect another. The most notable example are the 30 CRISPR spacers of *Metallosphaera sedula *(family Sulfolobaceae) that matched the genome of the *Acidianus *two-tailed virus [15,16], a virus also found to match 10 spacers present in another member of this family, *Sulfolobus solfataricus *strain P2. This is an indication that these two-tailed viruses may infect a broad-host range within Sulfolobaceae.

For spacers that match non mobility-associated ORFs in other species, one may also consider an alternative explanation, other than horizontal gene transfer. Spacers that may have caused autoimmunity could have led to a loss of the self-targeted gene, while orthologs of that gene persist in other related species. While this scenario can never be totally ruled out, one would expect that such a phenomenon will often rely on existence of related gene (or genes) in the organism that possessed the spacer that compensate for the lost homolog. We therefore looked for homologous genes by a BLASTX search of the gene matching the spacer against the proteome of the organism where the spacer originated, requiring E-value < E^-5^; > 66% sequence coverage and > 50% sequence similarity. Out of 26 spacers that had best matches in coding genes of other species (Additional File 6 Table S6, Additional File 8 Table S8), two encode conserved essential proteins that were presumably never lost (Orc1 and PCNA), 21 genes had no BLASTX matches, and only three had related proteins present. Thus, it appears that in general it is horizontal gene transfer, rather than gene loss driven by autoimmunity that underlies the accumulation of these spacers.

While the hits within archaea could be explained by previously known mechanisms of gene transfer in this domain (see above), hits matching eukaryotic or bacterial organisms are more difficult to explain. While *Pseudomonas putida *is known to harbor conjugative plasmids which can, in principle, be transmitted to archaeal cells, the gene that was recognized by *H. lacusprofundi *is chromosomal. Furthermore, *P. putida *is a freshwater bacterium while *H. lacusprofundi *is an obligate halophile, implying that exposure to *P. putida *DNA has probably been a rare event. The simplest possible explanation for these unusual spacers is that some archaea can take up naked DNA from the environment (i.e. natural competence), as was already demonstrated for thermophilic [17,18] and halophilic species (R. Thane Papke, submitted). These DNA uptake events can be non-specific, therefore introducing non-archaeal DNA into the cell, and may include bacterial, protozoan or even animal DNA. Although hits to eukaryotic (Additional File 11 Table S10) organisms had relatively low E-values, since these organisms have large genomes, the significance of these matches should be interpreted with caution. We therefore repeated these BLASTN searches, restricting the search to the genomes in question. Again, E-values smaller than 0.00001 were obtained (for alignments see Additional File 12 AddFigure 1), indicating that these matches are not due to some compositional enrichment in those genomes that increases likelihood of good matches to these spacers. Thus, a possible explanation of this phenomenon is that similarly to our own immune system, that is sometimes activated against food antigens, archaeal CRISPR/Cas can become activated during the uptake of DNA for nutritional purposes [see [19]]. Since eukaryotic sequence space is still poorly explored, and eukaryotic genomes contain a lot of non-coding material that can rapidly lose sequence similarity, it is possible that many more spacers are eukaryote-derived.

### Few archaeal spacers match metagenomic sequences

Counter to our expectations, there were only 49 matches (derived from 23 individual spacers) to metagenomic sequences, including viral metagenomes [20]. This is in contrast to the previous studies showing excellent correspondence between CRISPR spacers from metagenomes and viral communities from the same environment [8,21,22]. When focusing on haloarchaeal spacers and comparing them to the viral and microbial metagenomes of the San Diego saltern [20], we observed that a single spacer from *H. lacusprofundi *had six matches in the microbial metagenome, while another *H. lacusprofundi *spacer, a *H. marismortui *spacer and a *Halorhabdus utahensis *collectively yielded 11 matches in the viral metagenome from the same site (Additional File 13 Table S11). The haloarchaeal genomes in our dataset originate from archaea isolated from the Dead Sea, Israel (*H. marismortui*); Great Salt Lake, Utah, USA (*H. utahensis) *and Deep Lake, Antarctica (*H. lacusprofundi*). Thus, it is possible that few viral types are shared between these hyper-saline locations and the San Diego saltern, due to high levels of viral endemism, as suggested previously for bacteriophages [23].

### The location of spacer within the array can be used as an indicator for selection

**Figure 5:**
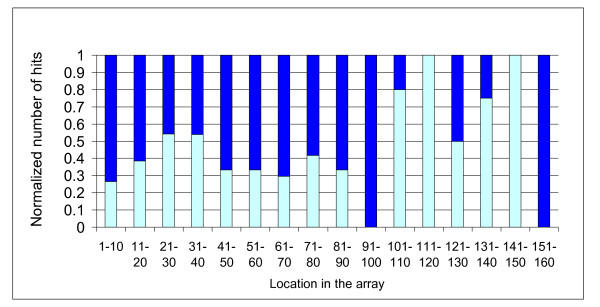
**The distribution of the normalized number of hits according to the location in the CRISPR array**. Light - viral hits; dark non-viral hits. Each result was normalized, dividing by the number of matches in each location bin.

Anti-viral defense has been highlighted as the major benefit of the CRISPR/Cas system to microorganisms. As can be expected, CRISPR spacers of one archaeal strain often match a virus known to infect another strain of the same species. For example, six spacers from *Methanothermobacter thermautotrophicus *strain Delta H match the Methanobacterium phage psiM2 [24], whose natural host is *M. thermautotrophicus *strain Marburg (Additional File 10 Dataset 1). New foreign DNA is integrated by the CRISPR/Cas system right next to the leader sequence, so older spacers tend to be at the distal end of the array. Newly acquired, recently integrated spacers have not had time for selection to operate on them, while older spacers can accumulate mutations unless they are under selection for retention. Thus, only spacers that are advantageous to the organism would be retained over long periods of time. Since anti-viral spacers are expected to be the most beneficial and therefore result in the strongest selection for retention, we examined the array location distribution of spacers that match archaeal viruses. As predicted, there was an enrichment for virus-matching spacers compared to the rest of the spacers in locations that are more distant from the CRISPR leader sequence (P = 0.024, Mann-Whitney-Wilcoxon test, Figures 4, 5). Surprisingly, there was also an enrichment in spacers with matches that fall outside the species which possesses the CRISPR array (P = 0.002, Mann-Whitney-Wilcoxon test, Figure 4), most of them matching viral sequences. Taken together, these findings can be interpreted as strong selection for spacers that provide resistance to viruses that are able to infect multiple hosts, or spacers that match several different viral types. These spacers can be thought of as the archaeal equivalent of broadly cross-protecting antibodies able to confer immunity against multiple strains of the same bacterial pathogen.

## Conclusions

The rapid nature of spacer acquisition and loss in CRISPR arrays makes spacers an important tool for the study of the ecology of archaeal species. This work demonstrates that there is much gene exchange within and between archaeal genera, and that anti-viral spacers, especially cross-protective ones, are preferentially retained. While the primary role of CRISPR/Cas systems appears to be to provide immunity against invading DNA, many spacers that are acquired can target random, presumably harmless, genes, just as vertebrate immune systems often recognize harmless antigens.

## Methods

### Spacer data

All available CRISPR spacers from archaea were downloaded from CRISPRdb http://crispr.u-psud.fr/crispr/ database [25] on 14.2.11. Following the exclusion of spacers defined by CRISPRdb as questionable, we retained 9219 out of 9424 spacers, derived from 78 archaeal genomes.

### Filtering of hits

Spacers were compared by NCBI BLASTN against the nr and env_nr nucleotide databases. BLASTN was preformed using a threshold of 0.001 for the E-value expectancy parameter, and setting the other parameters to default values. Self-hits (i.e. hits of spacers against themselves within the CRISPR array context were filtered out by removing all 100% identity hits where the organism was the same and the locus of the spacer and the hit sequence were the same, e.g. chromosome with chromosome or plasmid with plasmid).

We examined only hits that met all the following criteria: Coverage > = 0.5, Identity > = 0.85 and E-value < = E^-05^(Additional File 14 Dataset 2).

### Assignment of best hits

For all spacers that yielded hits that satisfied the above criteria, the best match was identified for each spacer individually. The best hit was judged as the sequence producing the maximal score according to the formula: 3 * (value of identity) + (value of coverage). In a few cases the best score according to this formula matched more than a single hit. When multiple best hits pointed to different functional classes (Figure 2), a hit of the spacer was counted once for each class.

For taxonomic assignment purposes, the best hits to cellular organisms and their plasmids allowed direct inference of origin. Hits to viruses were classified taxonomically by the known host species of the virus in question, according to the literature. Assignments of phylum, family, genus and species were according to NCBI taxonomy

For functional classification, spacers were classified by the type of mobile element they matched (virus or provirus, plasmid) or, in cases where they hit a chromosomal gene were classified as transposase gene, as integrase gene, as housekeeping if the gene in question had a putative cellular function, as a CRISPR gene if the gene in question had an annotation of CRISPR (*cas *genes/RAMP genes etc), as a mobility related gene (such as toxin/anti-toxin or DNA restriction/modification, on the chromosome) or as hypothetical if the gene was a hypothetical ORF or had a general function prediction only. The latter ORFs were also checked using BLASTP to confirm that no better functional annotation can be obtained by BLAST. This process was also performed when there was no annotation available for the target locus. Genes encoded on plasmids were classified as plasmids and were not classified as transposons (i.e. counted once) when the matches were to a plasmid-encoded transposase. In the absence of a coding gene hit, a hit was defined as being near a gene of a particular category (e.g. virus, archaeal host gene), if the NCBI BLAST annotation identified it being near such a feature (gene). Genes with functions that classified as housekeeping or hypothetical were also checked with regard to their gene neighborhood. In order to qualify as a housekeeping gene or hypothetical ORF with no viral feature, the gene had to be more than five genes apart, both upstream and downstream of any putative viral gene within the genome.

## Competing interests

The authors declare that they have no competing interests.

## Authors' contributions

AB participated in the design of the study, obtained and analyzed data, performed statistical analysis and wrote the manuscript. MNLW conceived of the study and participated in writing the manuscript. UG participated in the design of the study, obtained and analyzed data, performed statistical analysis and wrote the manuscript. All authors read and approved the final manuscript.

## Reviwer's comments

### Reviewer's report 1

W. Ford Doolittle, Dalhousie University.

### Reviewer comments

This is a nice little exercise in bioinformatics, showing that there is significant incorporation of non-mobile, non-viral genes as CRISPR spacers. Because the genomic databases are so skewed in terms of taxonomic inclusion, authors are wise to avoid getting very quantitative about their results.

It is interesting to wonder and important to know how these genes get into the recipients. The speculation that many archaea have the equivalent of nonspecific transformation systems seems unavoidable --- how else would they acquire eukaryotic DNA? The latter imports do raise further questions. Since there are relatively few eukaryotic genomes and since so much of so many of them is made up of non-coding sequences that would be expected to lose BLASTN detectability quickly, one might infer that most of the CRISPR sequences that match nothing are actually eukaryotic. Could that be so?

It also seems a bit of a no-brainer that most CRISPR phage hits are to phage infecting other (albeit congeneric) species. If the CRISPR is active, will it not ensure that phages that match it perfectly do not infect effectively? And where the hit is to a chromosomal gene not from the same species, cannot this simply mean that if it were, there would have been an autoimmunity problem?

Author's response:

"It is interesting to wonder and important to know how these genes get into the recipients. The speculation that many archaea have the equivalent of nonspecific transformation systems seems unavoidable --- how else would they acquire eukaryotic DNA? "

We agree with this comment, but just to make the message more pronounced we included the actual term competence in the text as follows:"... take up naked DNA from the environment (i.e. natural competence),"

The latter imports do raise further questions. Since there are relatively few eukaryotic genomes and since so much of so many of them is made up of non-coding sequences that would be expected to lose BLASTN detectability quickly, one might infer that most of the CRISPR sequences that match nothing are actually eukaryotic. Could that be so?

While we think that most CRISPR sequences come from under-represented (database-wise) archaeal viruses and not eukaryotes, we really liked that comment. Thus we added the sentence " Since eukaryotic sequence space is still poorly explored, and eukaryotic genomes contain a lot of non-coding material that can rapidly lose sequence similarity, it is possible that many more spacers are eukaryote-derived, "

It also seems a bit of a no-brainer that most CRISPR phage hits are to phage infecting other (albeit congeneric) species. If the CRISPR is active, will it not ensure that phages that match it perfectly do not infect effectively?

Nevertheless there are cases where a few strains of the same species have been sequenced, and there we do observe hits within the species. We realized that the figure legend did not state that "Within species matches are not shown.", and have added that sentence.

And where the hit is to a chromosomal gene not from the same species, cannot this simply mean that if it were, there would have been an autoimmunity problem?

Correct, and thus the adverb "Surprisingly" was removed from the sentence which now reads" Most spacers have best matches outside the specific CRISPR..."

### Reviewer's report: 2

John van der Oost, Wageningen University Brodt and co-workers report on an in silico analysis of CRISPR spacers in archaeal genomes. Whereas half of the spacers match with plasmids or viruses, a substantial number of matches have been detected with chromosomal genes of other organisms, mainly archaea. This observation has been interpreted as evidence for recombination events. The spacers with matches to viruses tend to be located more towards the 3' end of the CRISPR, indicative of the selective pressure to retain them. This study adds some details to the rapidly developing CRISPR field. The text requires serious polishing and more careful phrasing Comments

1) CRISPR/Cas is name for the defense system, CRISPR is the repetitive array;

when referring to gene(s), cas should be italics;

2) P.1, Background, line 3: should be: CRISPR arrays can be transcribed and processed into small crRNA molecules.

3) P.1, Background, line 4: should be: foreign nucleic acid for degradation.

4) P.1, Conclusions, line 3: should be: anti-viral and anti-plasmid defense.

5) P.2, Background, line 14-17: rephrase since archaeal systems have not been studied in enough detail - there may be a "seed; sequence there as well, as recently described for the E. coli system;

6) P.3, Results & Discussion, line 3-4: higher numbers of spacers - provide numbers in text

7) P.3, Results & Discussion, line 13: CRISPR should be: CRISPR-associated.

8) P.3, Results & Discussion, line 22-31: Mention the different types of recombination: Conjugation, Transfection, and Transformation.

9) P.3, Results & Discussion, last line: provide examples of CRISPR loci that reside on plasmid (add Suppl Table).

10) P.4, Results & Discussion, line 14-16: identity is not the only thing that matters, so does precence of PAM motif - include this in analysis and discussion;

11) P.5, Results & Discussion, line 3: substitute "organism" by "related virus";

Author's response:

1) CRISPR/Cas is name for the defense system, CRISPR is the repetitive array;

when referring to gene(s), cas should be italics;

Fixed

2) P.1, Background, line 3: should be: CRISPR arrays can be transcribed and processed into small crRNA molecules

Fixed, in both abstract and main text.

3) P.1, Background, line 4: should be: foreign nucleic acid for degradation.

Fixed

4) P.1, Conclusions, line 3: should be: anti-viral and anti-plasmid defense.

Fixed

5) P.2, Background, line 14-17: rephrase since archaeal systems have not been studied in enough detail - there may be a "seed; sequence there as well, as recently described for the E. coli system;

Rephrased to: "Archaeal CRISPR/Cas has been shown to confer almost 100% immunity in cases where spacers were identical to the target sequence, but partial matches also provide substantial immunity in archaea [12]. A short seed sequence that requires a perfect match has been recently discovered in bacteria [6], but whether such seed also exists in archaea, remains to be determined"

6) P.3, Results & Discussion, line 3-4: higher numbers of spacers - provide numbers in text.

Done

7) P.3, Results & Discussion, line 13: CRISPR should be: CRISPR-associated

Fixed

8) P.3, Results & Discussion, line 22-31: Mention the different types of recombination: Conjugation, Transfection, and Transformation.

Done

9) P.3, Results & Discussion, last line: provide examples of CRISPR loci that reside on plasmid (add Suppl Table).

Done

10) P.4, Results & Discussion, line 14-16: identity is not the only thing that matters, so does precence of PAM motif - include this in analysis and discussion;

We thank the reviewer for pointing that possibility, which we have overlooked. We have revised the text to: "The fact that this self-matching spacer is tolerated in this organism implies that either the proto-spacer associated motif (PAM) has been mutated in the chromosomal gene sequence rendering the gene immune [3] or that the chromosomal CRISPR/Cas system had become deactivated in this archaeon." We also performed some analysis, assuming that the Halomicrobium CRISPR/Cas is CRISPR group 1, like Haloarcula to which some of its *cas *genes show some similarity, we would expect the PAM to be (t/a)GG (based on Mojica et al., Microbiology 2009), but we find CCC in the chromosome instead, which should protect the gene from cleavage. This is very nice, but because of the assumptions/guesswork involved we would rather not include these results in the text.

11) P.5, Results & Discussion, line 3: substitute "organism" by "related virus";

Fixed

### Reviewer's report: 3

Christa Schleper, University of Vienna (nominated by J. Peter Gogarten, University of Connecticut)

The authors present an analysis of archaeal CRISPR spacers searching for their potential origin by DNA similarity searches in the public databases. As expected and shown in earlier studies, the authors demonstrate that most spacers are homologous to sequences of viruses or other mobile genetic elements, but they find also a considerable fraction of matches to classical chromosomally derived (or housekeeping) genes in other organisms. By superimposing the identified protospacers with taxonomic clusters, the authors give an overview of the potential sources of the archaeal spacer sequences which indicates potential routes of horizontal gene transfer.

The manuscript is very clearly and concisely written and it is inspiring. For example, I got the feeling that the frequent exchange of DNA among Sulfolobales, as often insinuated through conjugative and comparative genomic studies is somewhat reflected in the CRISPR world. It is also of interest to see, that several viruses in archaea might have a broader host range than expected.

Of course a potential chromosomal DNA transfer from eukaryotes, even humans, to archaea should be a rather rare event(!). However, I think that the authors should consider (and discuss) another potential mechanism to explain the general picture: Spacers, that were perhaps originally self-directed against the own genome and thus raised autoimmunity, might have caused selection for organisms, that have lost the respective target gene. In that case, the spacer, which remained, was originally derived from a gene of the own chromosome, but in today's blast searches the next best match appears to another (maybe even distantly related) organism. The authors might find out about this possibility, by checking if another orthologue of that gene is found in the chromosome of the spacer-carrying organism or not. Furthermore, I think it is of importance, to include in the analyses more than just the best BLAST matches. If e.g. a Methanococcus spacer matches a human sequence best, but the second best match with an almost identical e-value is to a bacterium, then I would be far less convinced of a eukaryotic-archaeal transfer.

Minor comments:

it is not possible for the reader to identify the spacers listed in tables 1-10. It would be helpfull to number the spacers of each organism and to link them to the additional data set, in which the spacer sequences are explicitly given (suppl. No. 12)

Figure 1: colour of Nanoarchaeota does not match with colour legend- Figure 4: not all loci have 150 spacers. Explain how this figure should be interpreted.

thermophilic, halophilic is misspelled

Authors response:

I think that the authors should consider (and discuss) another potential mechanism to explain the general picture: Spacers, that were perhaps originally self-directed against the own genome and thus raised autoimmunity, might have caused selection for organisms, that have lost the respective target gene. In that case, the spacer, which remained, was originally derived from a gene of the own chromosome, but in today's blast searches the next best match appears to another (maybe even distantly related) organism. The authors might find out about this possibility, by checking if another orthologue of that gene is found in the chromosome of the spacer-carrying organism or not.

This is an interesting suggestion, although one would still need to explain how the autoimmunity emerged in the first place, so exposure to foreign DNA that is similar is not altogether excluded. We performed the analysis suggested and added the following paragraph: " For spacers that match non mobility-associated ORFs in other species, one may also consider an alternative explanation, other than horizontal gene transfer. Spacers that may have caused autoimmunity could have led to a loss of the self-targeted gene, while orthologs of that gene persist in other related species. While this scenario can never be totally ruled out, one would expect that such a phenomenon will often rely on existence of related gene (or genes) in the organism that possessed the spacer that compensate for the lost homolog. We therefore looked for homologous genes by a BLASTX search of the gene matching the spacer against the proteome of the organism where the spacer originated, requiring E-value < E^-5^; > 66% sequence coverage and > 50% sequence similarity. Out of 26 spacers that had best matches in coding genes of other species (Additional Table S6, Additional Table S8), two encode conserved essential proteins that were presumably never lost (Orc1 and PCNA), 21 genes had no BLASTX matches, and only three had related proteins present. Thus, it appears that in general it is horizontal gene transfer, rather than gene loss driven by autoimmunity that underlies the accumulation of these spacers."

Furthermore, I think it is of importance, to include in the analyses more than just the best BLAST matches. If e.g. a Methanococcus spacer matches a human sequence best, but the second best match with an almost identical e-value is to a bacterium, then I would be far less convinced of a eukaryotic-archaeal transfer.

We have addressed this point by adding an additional dataset that includes all hits that passed our threshold - not just the best hits (Additional dataset 2). Regarding the hits to eukaryotes, there were no close matches to bacteria except for one case: the spacer that produced 29/29 nucleotide identity against two insect genomes also gave a 27/27 match to *hrpW *gene in *Pseudomonas viridiflava *a plant pathogen. Curiously this gene encodes an effector protein that elicits plant responses so it is imaginable that parts of it have been transferred from bacteria to eukaryotes or vice versa. In any case two fewer identical residues mean a weaker similarity by more than an order of magnitude.

Minor comments:

it is not possible for the reader to identify the spacers listed in tables 1-10. It would be helpfull to number the spacers of each organism and to link them to the suppl. data set, in which the spacer sequences are explicitely given (suppl. No. 12). To address this, we have now included the CRISPRdb reference of each spacer in all the additional tables, which have been re-done and are provided as Excel files. Thus, it is now easier to identify individual spacers.

Figure 1: colour of Nanoarchaeota does not match with colour legend. Fixed, Figure 4: not all loci have 150 spacers. Explain how this figure should be interpreted. We have re-phrased the legend of this figure, hope it is clearer now.

thermophilic, halophilic is misspelled. Fixed

## Supplementary Material

Additional file 1 **Table S1**. Spacers matching plasmids. Click here for file

Additional file 2 **Table S2**. Spacers matching viruses and proviruses, including non-coding sequences flanking a viral ORF. Click here for file

Additional file 3 **Table S3**. Spacers matching transposable elements, including non-coding sequences flanking a transposable element ORF. Click here for file

Additional file 4 **Table S4**. Spacers matching integrases, including non-coding sequences flanking an integrase. Click here for file

Additional file 5 **Table S5**. Spacers matching mobility associated genes, such as restriction -modification systems and toxin-anti-toxin systems, including non-coding sequences flanking such elements. Click here for file

Additional file 6 **Table S6**. Spacers matching housekeeping genes, including non-coding sequences flanking such genes. Click here for file

Additional file 7 **Table S7**. Spacers matching CRISPR-related genes, including non-coding sequences flanking CRISPRs. Click here for file

Additional file 8 **Table S8**. Spacers matching hypothetical genes, including non-coding sequences flanking such genes. Click here for file

Additional file 9 **Table S9**. CRISPR arrays located on plasmids. Click here for file

Additional file 10 **DataSet 1**. Best genomic matches of spacers identified in this study. Click here for file

Additional file 11 **Table S10**. Spacers matching eukaryotes. Click here for file

Additional file 12 **AddFigure 1**. BLASTN alignments of spacer sequences that match eukaryotic sequences. Click here for file

Additional file 13 **Table S11**. Spacers matching metagenomes. Click here for file

Additional file 14 **DataSet 2**. All significant genomic matches of spacers identified in this study, allowing multiple matches. Click here for file
