# Supplementary material for: CRISPR loci reveal networks of gene exchange in archaea
Source: Biol Direct. 2011 Dec 21;6:65. doi: 10.1186/1745-6150-6-65 (PMC3285040; doi:10.1186/1745-6150-6-65)
Supplement: Additional file 12 — AddFigure 1. BLASTN alignments of spacer sequences that match eukaryotic sequences. [file 1745-6150-6-65-S12.PDF]

BLAST alignments of spacer sequences that match eukaryotic sequences

1.

>ref|NM_001153675.1| Zea mays electron transporter (LOC100280754), mRNA
 gb|EU953574.1| Zea mays clone 1436416 electron transporter mRNA, complete cds
Length=1205

 GENE ID: 100280754 LOC100280754 | electron transporter [Zea mays]
(10 or fewer PubMed links)

Score = 54.0 bits (27), Expect = 2e-07
 Identities = 30/31 (97%), Gaps = 0/31 (0%)
 Strand=Plus/Plus

```
Query  2    CACCTCCGCCTCCCCCACCTCCACCTGCTCC  32
            |||||||||||||||| ||||||||||||||
Sbjct  193  CACCTCCGCCTCCCCCGCCTCCACCTGCTCC  223
```

2.
>gb|AC206355.3| Pongo abelii BAC clone CH276-439I18 from chromosome 5, complete
sequence
Length=192531

Score = 54.0 bits (27), Expect = 2e-07
 Identities = 30/31 (97%), Gaps = 0/31 (0%)
 Strand=Plus/Plus

```
Query  4       ATTTCTTCTATTATCTTTATTATTTCCTTCT  34
               |||||||||||||||||| ||||||||||||
Sbjct  172123  ATTTCTTCTATTATCTTTGTTATTTCCTTCT  172153
```

3.
>gb|AF363578.1| Homo sapiens ATPase H+ transporting lysosomal protein (ATP6C)
gene, complete cds; BAALC (BAALC) gene, complete cds, alternatively spliced; and frizzled-like protein 6 (FZD6) gene, complete
cds
Length=347253

Score = 54.0 bits (27), Expect = 6e-06
 Identities = 30/31 (97%), Gaps = 0/31 (0%)
 Strand=Plus/Minus

```
Query  3      TAAACAGATAATCAAAGAATTAAGAGAATTT  33
              ||||||||||| |||||||||||||||||||
Sbjct  86599  TAAACAGATAAGCAAAGAATTAAGAGAATTT  86569
```

4.

```
>dbj|AB007022.1|  Mesocricetus auratus gene for delta-sarcoglycan,
intron1, partial
sequence
Length=2725

Score = 54.0 bits (27),  Expect = 2e-09
 Identities = 27/27 (100%), Gaps = 0/27 (0%)
 Strand=Plus/Minus

Query  9    AAATTAATATTTGGTATAATTTTAAAG  35
            |||||||||||||||||||||||||||
Sbjct  449  AAATTAATATTTGGTATAATTTTAAAG  423
```

5.

```
>gb|AC023136.6|  Homo sapiens BAC clone RP11-497K21 from 4, complete
sequence
Length=185593

Score = 54.0 bits (27),  Expect = 5e-06
 Identities = 27/27 (100%), Gaps = 0/27 (0%)
 Strand=Plus/Plus

Query  2      ATGCTTTTAAATTAATATATTTTAATA  28
              |||||||||||||||||||||||||||
Sbjct  39083  ATGCTTTTAAATTAATATATTTTAATA  39109
```

6.

```
>gb|AF028262.1|AF028262  Hordeum chilense RAPD marker IAS-pHc6-4
Length=694

Score = 52.0 bits (26),  Expect = 9e-10
 Identities = 26/26 (100%), Gaps = 0/26 (0%)
 Strand=Plus/Plus

Query  7    TATTTTTATTTTATCTTTATCTATCA  32
            ||||||||||||||||||||||||||
Sbjct  375  TATTTTTATTTTATCTTTATCTATCA  400
```

7.

```
>gb|AE014298.4|  Drosophila melanogaster chromosome X, complete
sequence
Length=22422827

Score = 58.0 bits (29),  Expect = 4e-08
 Identities = 29/29 (100%), Gaps = 0/29 (0%)
 Strand=Plus/Plus

Query  11       CACCGCCACCGCCTCCGCCACCACCACCT  39
                |||||||||||||||||||||||||||||
Sbjct  2818103  CACCGCCACCGCCTCCGCCACCACCACCT  2818131
```

7b. (equal score best hit)

```
>ref|XM_001604781.1|  PREDICTED: Nasonia vitripennis similar to
CG32705-PA (LOC100121238),
mRNA
Length=2229

 GENE ID: 100121238 LOC100121238 | similar to CG32705-PA [Nasonia
vitripennis]
(10 or fewer PubMed links)

Score = 58.0 bits (29),  Expect = 2e-09
 Identities = 29/29 (100%), Gaps = 0/29 (0%)
 Strand=Plus/Minus

Query  11    CACCGCCACCGCCTCCGCCACCACCACCT  39
             |||||||||||||||||||||||||||||
Sbjct  2047  CACCGCCACCGCCTCCGCCACCACCACCT  2019
```

8.

```
>gb|AC108910.18|  Mus musculus chromosome 18, clone RP24-550O15,
complete sequence
Length=206978

Score = 56.0 bits (28),  Expect = 1e-06
 Identities = 28/28 (100%), Gaps = 0/28 (0%)
 Strand=Plus/Plus

Query  9       AGAGAATAGAGAATAGAGAACAGAGAAC  36
               ||||||||||||||||||||||||||||
Sbjct  128741  AGAGAATAGAGAATAGAGAACAGAGAAC  128768
```

9.

```
>ref|XM_632242.1|  Dictyostelium discoideum AX4 WD40 repeat-
containing protein (cdh1)
mRNA, complete cds
Length=2265

 GENE ID: 8626032 cdh1 | WD40 repeat-containing protein
[Dictyostelium discoideum AX4] (10 or fewer PubMed links)

Score = 54.0 bits (27),  Expect = 3e-08
 Identities = 30/31 (97%), Gaps = 0/31 (0%)
 Strand=Plus/Minus

Query  2    GTGGTAGTGGTGGTAGTTGTTGTTGTTGTAG  32
            ||||||||||||||||||||||||| |||||
Sbjct  341  GTGGTAGTGGTGGTAGTTGTTGTTGGTGTAG  311
```
